# Syndrome of Inappropriate Antidiuretic Hormone Secretion and Thrombotic Microangiopathy as Paraneoplastic Syndromes Complicating BRCA2-Mutated Metastatic Prostate Cancer

**DOI:** 10.7759/cureus.101249

**Published:** 2026-01-10

**Authors:** Nidha Shapoo, Carlos Izaguirre, Abdul Rehman, Joseph Mattana, Vladimir Gotlieb

**Affiliations:** 1 Internal Medicine, New York Medical College, Metropolitan Hospital Center, New York, USA; 2 Oncology, New York Medical College, Metropolitan Hospital Center, New York, USA

**Keywords:** aggressive subtype, brca2 mutation, hematologic abnormalities, metastatic prostate cancer, siadh, thrombotic microangiopathy

## Abstract

Metastatic prostate cancer with a BRCA2 mutation is associated with aggressive clinical behavior and poor outcomes with standard systemic therapy. While the BRCA2 mutation predicts response to PARP inhibitors and platinum agents, its association with paraneoplastic syndromes is not well described. We report a 72-year-old male who presented with altered sensorium with severe hyponatremia who was diagnosed with syndrome of inappropriate antidiuretic hormone secretion (SIADH) in the context of newly diagnosed metastatic prostate adenocarcinoma. He was treated with free water restriction, hypertonic saline, and triplet systemic therapy (docetaxel, androgen deprivation, and darolutamide) and discharged in stable condition. After one year of disease remission, he relapsed with bone marrow metastases, confirming the BRCA2 mutation, and presented with thrombotic microangiopathy (TMA). The patient was managed with chemotherapy along with blood support, with clinical improvement; however, he succumbed to the disease within two months. While SIADH and TMA have been individually reported in prostate cancer, their sequential occurrence in a BRCA2-mutated setting is unique. Management of these complications requires addressing the underlying cancer with definitive treatment. The case highlights the vigilance for atypical paraneoplastic manifestations, the need for early genomic testing, and the exploration of novel therapeutic strategies in BRCA2-driven prostate cancer.

## Introduction

Metastatic prostate cancer (mPC) is an aggressive disease with a 5-year survival rate of 30% and is the second leading cause of cancer-related death in men [1]. According to the Surveillance, Epidemiology, and End Results (SEER) study, the age-adjusted incidence rate of mPC increased from 11.58 per 100,000 in 2004 to 17.30 per 100,000 in 2018 in men aged 45-74 [2]. Metastatic castrate resistant prostate (mCRPC) cancer is the most aggressive form, where patients develop resistance to androgen deprivation therapy [3]. The heterogeneity in outcomes for mCRPC is suggested to be due to the underlying molecular genetic features. The most essential genomic mutations are alterations in DNA damage repair genes, including those involved in DNA homologous recombination repair (HRR), found in up to 30% of mPC. The most common HRR alterations in mCRPC are found in BRCA2, seen in approximately 3-15% of patients [4]. BRCA2-mutated prostate cancers have a more aggressive course with a poor response to chemotherapy and hormonal agents; however, this mutation predicts response to poly ADP-ribose polymerase (PARP) inhibitors [5,6]. While BRCA2-mutated prostate cancer is recognized for its aggressive clinical course, paraneoplastic syndromes and thrombotic microangiopathy (TMA) have not been well-described in this subset. Here, we report a rare case of BRCA2-mutated metastatic prostate cancer complicated by the sequential development of syndrome of inappropriate antidiuretic hormone secretion (SIADH) and TMA, highlighting an unusual paraneoplastic scenario and a fatal course.

## Case presentation

**Figure 1:**
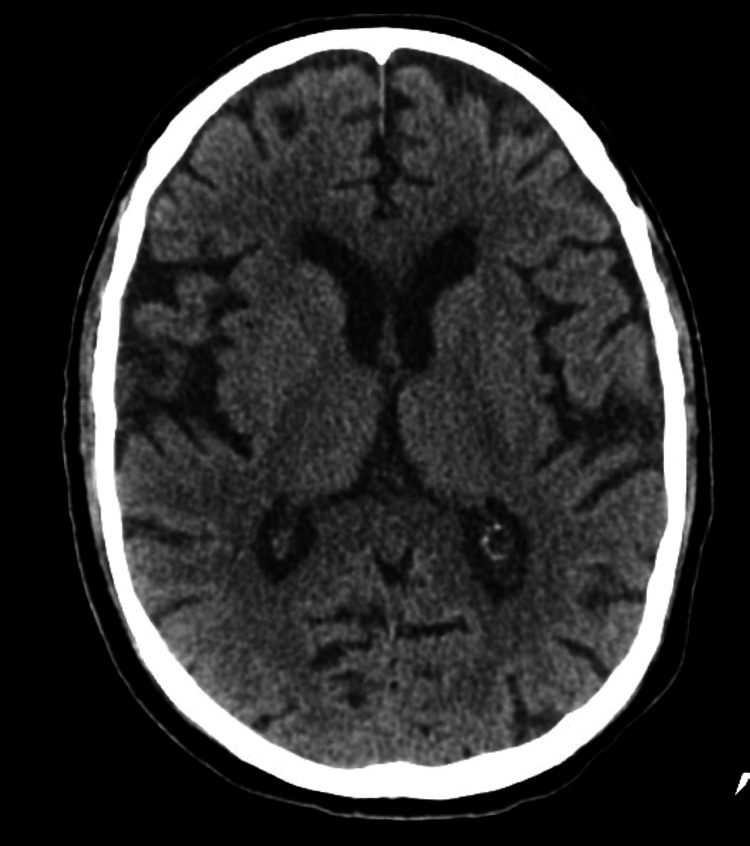
CT scan of the head showing no gross abnormality

A 72-year-old man with hypertension presented to the hospital with altered sensorium. He was drowsy, with an otherwise unremarkable physical examination, including no focal neurological deficits. His blood pressure was 130/78 mmHg, heart rate 80/min, temperature 37.5 °C, and oxygen saturation (SpO2) 97% on room air. The laboratory investigations revealed hyponatremia with urine osmolality higher than the serum osmolality (Table 1). Computed tomography (CT) of the head did not show any abnormality (Figure 1).

**Table 1:** Laboratory investigations

Components	Result	Normal Reference Range
Hemoglobin	13 g/dL	13 to 16 g/dL
Total Leukocyte Count	4500/microL	4000-11000 microL
Platelet Count	156000/microL	150000- 450000 microL
Serum Sodium	126 mEq/L	135 – 145 mEq/L
Serum Potassium	3.8 mEq/L	3.5 - 5.5 mEq/L
Blood Urea Nitrogen	5 mg/dL	6-20 mg/dl
Serum Creatinine	0.7 mg/dL	0.3 to 1 mg/dL
Blood Glucose	154 mg/dL	70-100 mg/dL
Serum Osmolality	245 mOsm/kg	270-290 mOsm/kg
Urine Osmolality	468 mOsm/kg	50-800 mOsm/kg
Urine Sodium	75 mEq/L	20-200 mEq/L

**Figure 2:**
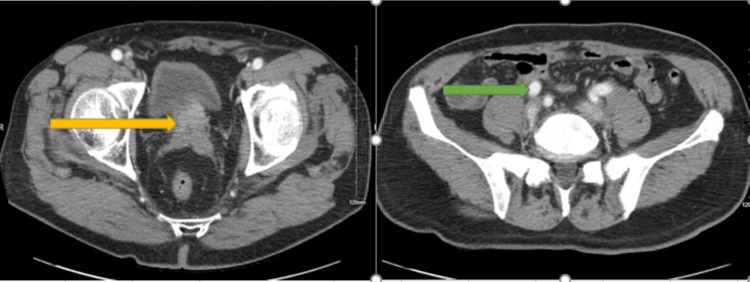
CT abdomen and pelvis with contrast showing an enlarged prostate (yellow arrow) and retroperitoneal lymphadenopathy (green arrow)

CT of the abdomen and pelvis with contrast revealed an enlarged prostate, retroperitoneal lymphadenopathy, and multiple osteoblastic bone lesions suggestive of metastatic prostate cancer (Figure 2).

**Figure 3:**
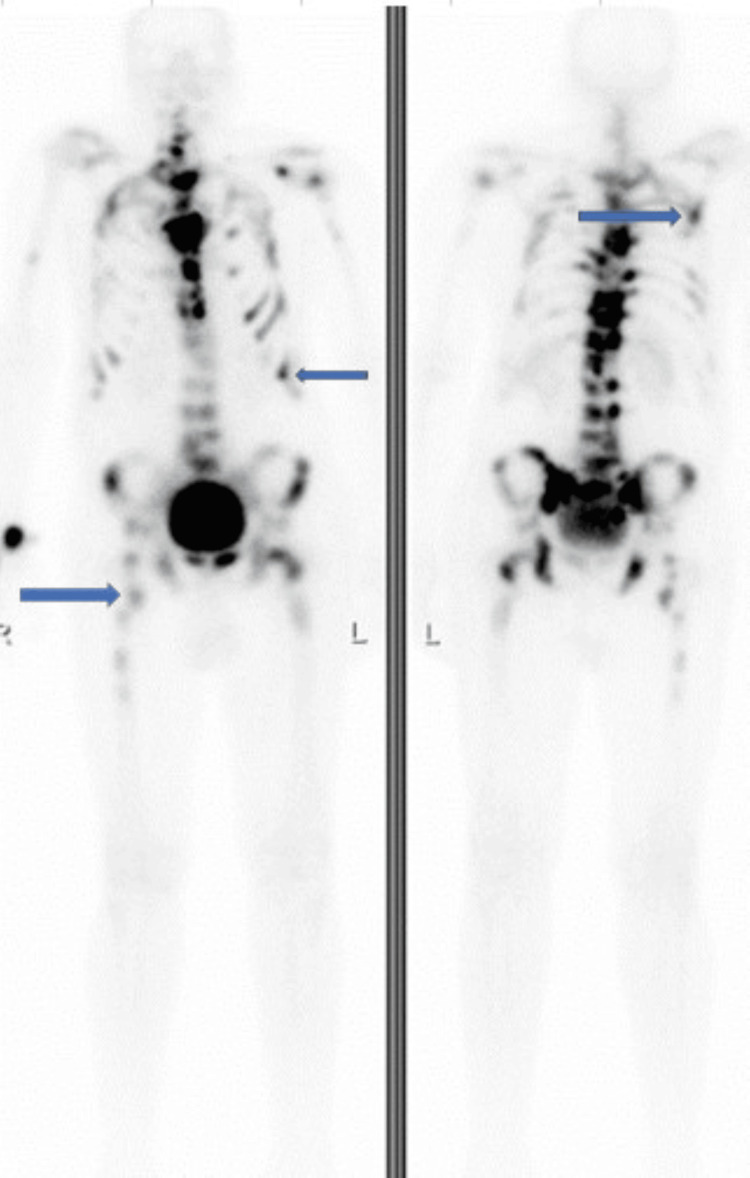
Whole body bone scan showing widespread metastasis involving the axial skeleton, including multiple bilateral ribs and proximal femurs bilaterally (blue arrows).

Whole body bone scan confirmed widespread metastasis involving the axial skeleton, including multiple bilateral ribs and proximal femurs bilaterally (Figure 3).

Prostate-specific antigen (PSA) was markedly elevated at 521 ng/mL. Urology was consulted, and a prostate biopsy was done, which revealed acinar adenocarcinoma with a Gleason score of 9. The cancer was high-risk, with extra prostatic extension and perineural invasion. Whole body bone scan confirmed widespread metastatic lesions in the axial skeleton, including multiple bilateral ribs and proximal femurs bilaterally. Given his hypotonic hyponatremia in the setting of apparent euvolemia, inappropriately concentrated urine, normal urine sodium, and absence of other factors that may cause hyponatremia, a diagnosis of SIADH was made.

The patient was treated with fluid restriction, furosemide, and a 3% NaCl solution, resulting in a gradual increase in serum sodium to 135 mEq/L. Triplet therapy consisting of docetaxel, androgen deprivation therapy (bicalutamide followed by Leuprolide), and darolutamide was started, and the patient was discharged in stable condition for outpatient follow-up. He went into remission after 6 cycles of docetaxel with normalization of PSA to 2.95 ng/mL, and he was continued on leuprolide and darolutamide with three-monthly follow-ups.

After six months of chemotherapy, the patient was admitted with generalized weakness and hematuria. The patient was seen three months before this admission without symptoms and with a normal PSA. The laboratory investigations revealed anemia, thrombocytopenia, elevated reticulocyte count, elevated lactate dehydrogenase, low serum haptoglobin, indirect hyperbilirubinemia, elevated aspartate transaminase, elevated creatinine, hyponatremia, and increased PSA (Table 2).

**Table 2:** Laboratory investigations PSA: prostate-specific antigen

Components	Result	Normal Reference Range
Hemoglobin	8.7 g/dL	13 to 16 g/dL
Total leukocyte count	5920/microL	4000-11000 microL
Platelet count	25000/microL	150000- 450000 microL
Serum sodium	129 mEq/L	135 – 145 mEq/L
Serum potassium	4 mEq/L	3.5 - 5.5 mEq/L
Blood urea nitrogen	20 mg/dL	6-20 mg/dl
Serum creatinine	1.6 mg/dL	0.3 to 1 mg/dL
Blood glucose	120 mg/dL	70-100 mg/dL
Total bilirubin	1.8 mg/dl	0.2-1.2 mg/dL
Direct bilirubin	0.4 mg/dL	0.3 – 0.4 mg/dl
Indirect bilirubin	1.4 mg/dL	0.2 to 0.6 mg/dL
Aspartate transaminase	181U/L	8-45 U/L
Alanine transaminase	12 U/L	7- 40 U/L
Alkaline phosphatase	919U/L	40-129 IU/L
Serum lactate dehydrogenase	1738 IU/L	130-220 IU/L
Reticulocytes	4.91%	0.5-2 %
Serum haptoglobin	< 20 mg/dL	40 to 200 mg/dL
Serum PSA	1332 ng/mL	< 4ng/ml

**Figure 4:**
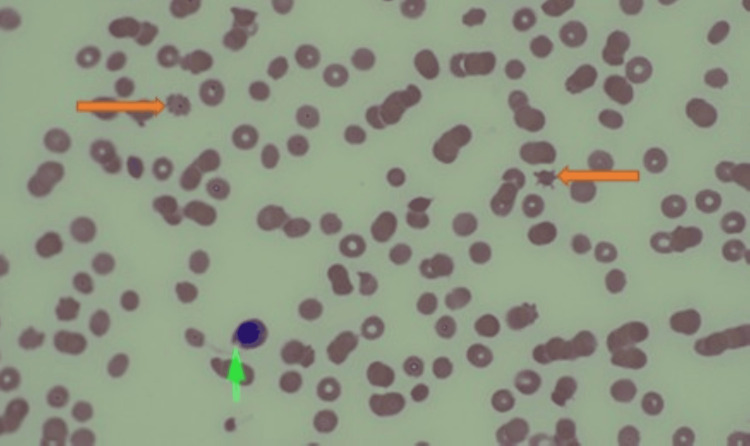
Peripheral smear shows nucleated red blood cells (green arrow) and schistocytes (orange arrows), suggesting TMA TMA: thrombotic microangiopathy

The peripheral smear revealed schistocytes, nucleated RBCs, and immature granulocytes, suggesting TMA (Figure 4).

**Figure 5:**
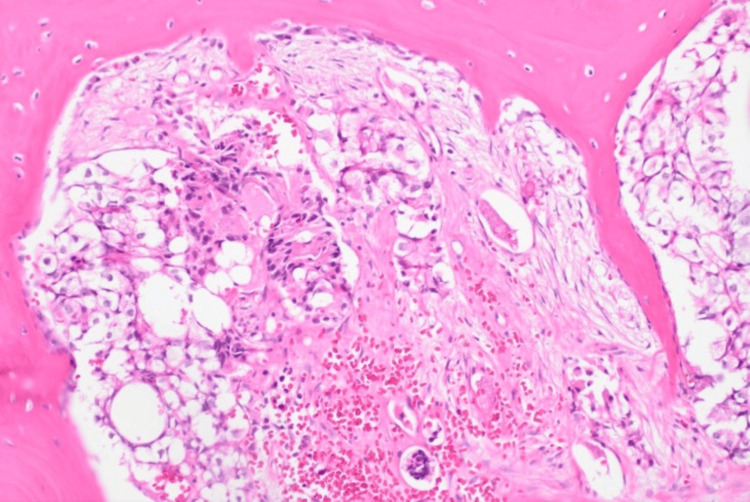
Bone marrow biopsy shows metastatic prostate carcinoma H&E, 100x

The PSA of 1,332 ng/ml was strongly suggestive of disease progression. A bone marrow biopsy confirmed metastatic prostate adenocarcinoma and revealed a BRCA2 mutation (p.(trp1692MetfsTer3) (Figure 5).

The patient’s PLASMIC score was 4, and ADAMTS13 activity was normal (81%), ruling out thrombotic thrombocytopenic purpura. The patient was treated with five cycles of docetaxel/carboplatin along with blood product support. Hyponatremia was managed with fluid restriction. The patient showed marked clinical and laboratory improvement with normalization of hematological abnormalities, liver enzymes, renal functions, and hyponatremia. The patient was discharged on a combination of olaparib, abiraterone, and leuprolide. Two months later, the patient relapsed and died.

## Discussion

Paraneoplastic syndromes are rare in prostate cancer. SIADH in prostate cancer has been presented in case reports, with some reports showing an association with poorly differentiated or small cell histology resulting from the ectopic secretion of ADH (antidiuretic hormone or vasopressin) from tumor cells. SIADH may be the presenting manifestation of cancer or develop during disease progression. Water restriction is the standard initial treatment, though the primary treatment lies in the control of the underlying cancer [7-9]. Abe et al. reported SIADH and humoral hypercalcemia of malignancy in a patient with prostate cancer and pulmonary metastases [9]. The patient was treated with water restriction, hypertonic saline, tolvaptan, and radiation to the prostate due to local symptoms. The serum sodium improved from 114 mEq/L at presentation to 135 mEq/L at discharge [9]. In our patient, SIADH was the initial presentation of cancer and developed at the time of progression. Management with water restriction and treatment of the underlying cancer led to significant improvement in the patient’s clinical condition and electrolyte balance.

TMA refers to disorders characterized by microangiopathic hemolytic anemia, thrombocytopenia, and organ dysfunction due to thrombus formation affecting small or larger vessels. The most recognized forms of TMA are thrombotic thrombocytopenic purpura (TTP) and hemolytic uremic syndrome (HUS). Cancer-associated TMA has been reported to range from 6% to 15%, seen most often in patients with metastatic breast, gastric, and lung cancer [10]. The exact mechanism by which solid tumors induce TMA is complex and multifactorial. The clinical spectrum of TMA may vary widely from asymptomatic abnormal laboratory tests to acute, severe, potentially life-threatening forms due to massive microvascular occlusion. Patients with TMA often present with fatigue, pallor, jaundice, easy bruising, bleeding complications, and acute kidney injury. The diagnosis typically involves a combination of anemia, thrombocytopenia, a peripheral blood smear showing schistocytes, elevated lactate dehydrogenase, non-conjugated bilirubin, and reticulocyte count, and low haptoglobin suggestive of hemolysis. A normal ADAMTS-13 activity rules out TTP. Cancer-associated TMA is mainly associated with adenocarcinoma histology and with advanced disease. The treatment involves treating the underlying cancer, as plasmapheresis, steroids, or other immunosuppressive agents used in TTP have no beneficial role. Complement inhibitors, such as eculizumab, have shown promise in treating HUS and chemotherapy-induced TMA. Cancer-associated TMA is a rare condition associated with poor prognosis [10,11]. TMA associated with prostate cancer is rare and exists as case reports [12,13]. Newton et al. described a patient with prostate cancer who was diagnosed with TMA and found to have recurrent prostate cancer with mediastinal lymphadenopathy. The patient underwent two sessions of hemodialysis, blood and platelet transfusions, and a dose of eculizumab. He was started on hormonal treatment for prostate cancer. The patient improved and was discharged in stable condition [12]. Some chemotherapeutic agents, such as gemcitabine, cisplatin, carboplatin, mitomycin, bleomycin, and, rarely, docetaxel, have been reported to cause TMA, which can occur between one day and eight months [14]. Yu O et al. reported a patient with advanced prostate cancer with pulmonary tumor thrombotic microangiopathy who responded temporarily to docetaxel chemotherapy [15]. The fact that TTP in our patient also responded to docetaxel-based chemotherapy suggests that docetaxel may be effective in such cases.

A BRCA2 mutation is strongly associated with aggressive disease across the continuum. The patients present more often with high-grade tumors, nodal and distant metastases, and have inferior survival compared to BRCA2-negative tumors. In advanced disease, BRCA2 mutations predict earlier castration resistance and poor response to standard hormone therapy and chemotherapy, with real-world data confirming shorter survival. Pathologically, BRCA2 alterations correlate with intraductal/cribriform histology and genomic features, such as BRCA2/RB1 co-loss, that drive aggressive clinical behavior [4,5]. Therapeutically, BRCA2 predicts sensitivity to PARP inhibitors and platinum, explaining our patient’s transient response to platinum-based chemotherapy and Olaparib. Nonetheless, responses are often short-lived in this aggressive subtype [6].

Though our patient improved with supportive care and chemotherapy, hormone therapy, and olaparib for prostate cancer, the remission was short-lasting, and he succumbed to the disease after two months.

During both admissions of our patient, the hyponatremia and TMA improved after definitive treatment of cancer, and the patient had normal sodium values on follow-ups. There are no specific guidelines for the follow-up of such patients, but our patient was being closely monitored with three-monthly follow-ups.

Our case adds novelty to the occurrence of SIADH at presentation and cancer-associated TMA at progression. Both have been rarely described in prostate cancer, typically with advanced disease, but not explicitly linked to BRCA2. We hypothesize that tumor burden and marrow infiltration in BRCA2-driven disease created a pro-inflammatory, pro-thrombotic milieu, predisposing to these syndromes.

## Conclusions

To our knowledge, this is the first reported case of BRCA2-mutated metastatic prostate cancer complicated by SIADH and TMA. There is no specific treatment, but addressing the underlying cancer may result in the resolution of these complications. BRCA2 status is a key driver of aggressive biology in metastatic prostate cancer. The unique combination of SIADH and TMA observed here underscores the need for clinicians to consider atypical paraneoplastic and microangiopathic phenomena in advanced disease, as well as the importance of early molecular testing, timely BRCA-directed therapy, and trial-based strategies.
